# MiCId GUI: The Graphical User Interface for MiCId, a Fast Microorganism Classification and Identification Workflow with Accurate Statistics and High Recall

**DOI:** 10.1089/cmb.2023.0149

**Published:** 2024-02-12

**Authors:** Aleksey Ogurtsov, Gelio Alves, Alex Rubio, Brendan Joyce, Björn Andersson, Roger Karlsson, Edward R.B. Moore, Yi-Kuo Yu

**Affiliations:** ^1^ National Center for Biotechnology Information, National Library of Medicine, National Institutes of Health, Bethesda, Maryland, USA.; ^2^ Bioinformatics Core Facility, Sahlgrenska Academy, University of Gothenburg, Gothenburg, Sweden.; ^3^ Department of Infectious Diseases, Sahlgrenska Academy, University of Gothenburg, Gothenburg, Sweden.; ^4^ Nanoxis Consulting AB, Gothenburg, Sweden.; ^5^ Culture Collection University of Gothenburg, Sahlgrenska Academy, University of Gothenburg, Sweden.

**Keywords:** antimicrobial-resistant proteins, biomass estimation, graphical user interface, mass-spectrometry-based proteomics, microorganism identification

## Abstract

Although many user-friendly workflows exist for identifications of peptides and proteins in mass-spectrometry-based proteomics, there is a need of easy to use, fast, and accurate workflows for identifications of microorganisms, antimicrobial resistant proteins, and biomass estimation. Identification of microorganisms is a computationally demanding task that requires querying thousands of MS/MS spectra in a database containing thousands to tens of thousands of microorganisms. Existing software can't handle such a task in a time efficient manner, taking hours to process a single MS/MS experiment. Another paramount factor to consider is the necessity of accurate statistical significance to properly control the proportion of false discoveries among the identified microorganisms, and antimicrobial-resistant proteins, and to provide robust biomass estimation. Recently, we have developed Microorganism Classification and Identification (MiCId) workflow that assigns accurate statistical significance to identified microorganisms, antimicrobial-resistant proteins, and biomass estimation. MiCId's workflow is also computationally efficient, taking about 6–17 minutes to process a tandem mass-spectrometry (MS/MS) experiment using computer resources that are available in most laptop and desktop computers, making it a portable workflow. To make data analysis accessible to a broader range of users, beyond users familiar with the Linux environment, we have developed a graphical user interface (GUI) for MiCId's workflow. The GUI brings to users all the functionality of MiCId's workflow in a friendly interface along with tools for data analysis, visualization, and to export results.

## 1. INTRODUCTION

Tandem mass-spectrometry-based proteomics is a useful tool for fast and accurate identifications of microorganisms and antibiotic resistant (AR) proteins, and estimation of sample's microorganismal-biomass composition. Fast and accurate identification/diagnosis of pathogenic bacteria along with the identification of AR proteins; hence, proper antibiotic treatment is among the most important to fight infections, to reduce the spread of antibiotic resistance, and to increase patients' survival rate (Tian et al., 2019). Obtaining trustworthy biomass estimates on the other hand are essential for microbial community structure analyses that arise in almost every microbiome study (Cani, 2018; Franzosa et al., 2015). Consequently, versatile tandem mass spectrometry-based proteomics data analysis software and pipelines that are easy to use, fast, accurate, and capable of performing identifications of microorganisms, AR proteins, and biomass estimation are welcome (Beyter et al., 2018). For this purpose, we have developed Microorganism Classification and Identification (MiCId)'s workflow.

MiCId's workflow can perform identifications of microorganisms, AR proteins, and biomass estimation in about 6–17 minutes using computer resources that are available in most laptop and desktop computers. MiCId's workflow has been extensively tested; it was shown to compute accurate microorganismal-biomass and statistical significance, *E*-values, to identified microorganisms and AR proteins. When employing the recommended *E*-value cutoff of 0.01, it has a recall of 95% at the species level identification with the proportion of false discoveries controlled below 5%. In terms of AR protein identification, MiCId's workflow has a sensitivity value around 85% (with a lower bound at about 72%) and a precision greater than 95% in identifying antibiotic resistance proteins (Alves and Yu, 2020; Alves et al., 2022; Alves et al., 2018; Kondori et al., 2021). The graphical user interface (GUI) brings to users all the functionality of MiCId's workflow in an easy-to-use interface along with several tools. Here we will briefly introduce MiCId's GUI and we provide detailed instructions on how to use it in the user's manual available with the GUI.

## 2. MICID'S GUI ARCHITECTURE

**FIG. 1.**
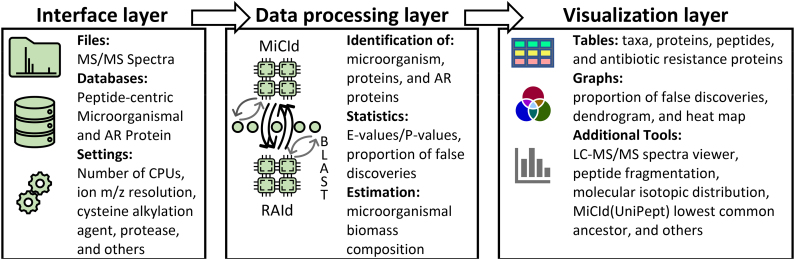
MiCId's GUI three function layers. The interface layer includes executable files that create databases, processes MS/MS spectra files, and initializes query parameters. The data processing layer is where all the work gets done; it includes the MiCId, RAId, BLAST programs, and a few scripts to process data and communicate between programs. The visualization layer provides tools for data analysis and visualization with the option of exporting figures and tables. GUI, graphical user interface; MiCId, Microorganism Classification and Identification; MS/MS, tandem mass-spectrometry.

MiCId's workflow has three functional layers, as displayed in Figure 1. The interface layer includes executable files that create databases and process tandem mass-spectrometry (MS/MS) spectra files. The data processing layer includes MiCId (Alves et al., 2022), RAId (Alves et al., 2007), and BLAST (Altschul et al., 1990) programs that have been implemented using C++ 17 programming language to optimize execution time. The visualization layer includes C/C++ and Perl (version 5.16.3) servicing scripts. The interface and visualization layers of the GUI are coded using Eclipse (Eclipse Foundation) Java Integrated Development Environment (IDE). The Java libraries used are the standard java.awt and the javax.swing provided by Oracle (JavaTM Platform Standard Ed. 7), and three class libraries downloaded from the Git repository: JFreeChart (https://github.com/jfree/jfreechart) for generating charts, iText 5 (https://github.com/itext/itextpdf) for generating PDF files, and hierarchical-clustering-java (https://github.com/lbehnke/hierarchical-clustering-java repository) for generating dendrograms. MiCId's GUI was tested under (1) Windows 10 Enterprise, (2) CentOS Linux release 7.9.2009, (3) Red Hat Enterprise Linux Server release 7.9, and (4) Ubuntu release 18.04.3.

## 3. DATA ANALYSIS USING MICID'S GUI

Once the GUI is installed, it offers user the choice of testing MiCId's workflow via the Test tab; instructions on how to use the GUI is also available to users via the User Guide tab. Both tabs are located under the MiCId tab in the main interface.

The first step is to create a microorganismal database and an antibiotic resistance protein database. To create a microorganismal database, the user needs to specify the taxonomy identifier of taxa to be included in the database using the Create organismal database tab. Taxonomy identifier used are taken from the National Center for Biotechnology Information (NCBI) database. MiCId GUI will then automatically download the protein sequences of all the taxa specified from the NCBI database along with taxonomical information and construct a microorganismal database. For the AR protein database, users can select from the Create AMR database tab among three established AR databases: the Comprehensive Antibiotic Resistance Database (CARD), Resfinder, and the National Database of Antibiotic Resistant Organisms (NDARO), or using their own customized AR protein database in a FASTA file. The second step is to specify a single or multiple experimental MS/MS files. MiCId GUI accepts MS/MS files of the following types: mzML, mzXML, mgf, and Thermo Scientific RAW. The third step is to set up query parameters such as number of logical cores, protease, cysteine reducing agent, taxa selection, precursor-ion and product-ion resolutions, and so on. The final step is to press the Submit job button and the code within the GUI and MiCId's workflow will perform the identification of microorganisms, identification of proteins, identification of AR proteins, and estimation of sample microorganismal-biomass composition, all with a single command without further user interventions.

New submitted jobs can be monitored and controlled by the user using the submitted jobs monitoring queue viewer, which allows users to check the status of submitted jobs, cancel, remove, restart, view completed job results, and more. For reproducibility and record-keeping, the GUI offers a Save Settings tab that will save all the settings used to execute a specific job and it creates a log file containing detailed information for each submitted job.

The GUI has the functionality for data plotting, visualizing results, and exporting tables and figures for publications. It also includes MS/MS and MS spectra viewers and several tools for computing and studying peptide fragmentation, peptide/protein isotopic distributions (Alves et al., 2014), lowest common ancestor algorithm for proteotyping biomarker designing, etc.

## SOFTWARE AVAILABILITY

MiCId's GUI version (v.11.11.2021) is freely available at https://www.ncbi.nlm.nih.gov/CBBresearch/Yu/downloads.html

## References

[B1] Altschul SF, Gish W, Miller W, et al. Basic local alignment search tool. J Mol Biol 1990;215(3):403–410; doi: 10.1016/S0022-2836(05)80360-2 2231712

[B2] Alves G, Ogurtsov A, Karlsson R, et al. Identification of antibiotic resistance proteins via micid's augmented workflow. A mass spectrometry-based proteomics approach. J Am Soc Mass Spectr 2022;33(6):917–931; doi: 10.1021/jasms.1c00347 PMC9164240 35500907

[B3] Alves G, Ogurtsov AY, Yu Y-K. Molecular isotopic distribution analysis (midas) with adjustable mass accuracy. J Am Soc Mass Spectr 2014;25(1):57–70; doi: 10.1007/s13361-013-0733-7 PMC3880471 24254576

[B4] Alves G, Ogurtsov AY, Yu Y-K. Raid_dbs: Peptide identification using database searches with realistic statistics. Biol Direct 2007;2(1):25; doi: 10.1186/1745-6150-2-25 17961253 PMC2211744

[B5] Alves G, Wang G, Ogurtsov AY, et al. Rapid classification and identification of multiple microorganisms with accurate statistical significance via high-resolution tandem mass spectrometry. J Am Soc Mass Spectr 2018;29(8):1721–1737; doi: 10.1007/s13361-018-1986-y PMC6061032 29873019

[B6] Alves G, Yu Y-K. Robust accurate identification and biomass estimates of microorganisms via tandem mass spectrometry. J Am Soc Mass Spectr 31(1):85–102; doi: 10.1021/jasms.9b00035 PMC10501333 32881514

[B7] Beyter D, Lin MS, Yu Y, et al. Proteostorm: An ultrafast metaproteomics database search framework. Cell Syst 2018;7(4):463–467.e6; doi: 10.1016/j.cels.2018.08.009 30268435 PMC6231400

[B8] Cani PD. Human gut microbiome: Hopes, threats and promises. Gut 2018;67(9):1716–1725; doi: 10.1136/gutjnl-2018-316723 29934437 PMC6109275

[B9] Franzosa EA, Hsu T, Sirota-Madi A, et al. Sequencing and beyond: integrating molecular ‘omics' for microbial community profiling. Nat Rev Microbiol 2015;13(6):360–372; doi: 10.1038/nrmicro3451 25915636 PMC4800835

[B10] Kondori N, Kurtovic A, Piñeiro-Iglesias B, et al. Mass spectrometry proteotyping-based detection and identification of Staphylococcus aureus, Escherichia coli, and Candida albicans in blood. Front Cell Infec Microbiol 2021;11:634215; doi: 10.3389/fcimb.2021.634215 34381737 PMC8350517

[B11] Tian L, Zhang Z, Sun Z. Antimicrobial resistance trends in bloodstream infections at a Large Teaching Hospital in China: A 20-Year Surveillance Study (1998–2017). Antimicrob Resist Infect Control 2019;8(1):86; doi: 10.1186/s13756-019-0545-z 31161033 PMC6540536

